# The effects of time valuation in cancer optimal therapies: a study of chronic myeloid leukemia

**DOI:** 10.1186/s12976-019-0106-4

**Published:** 2019-05-28

**Authors:** Pedro José Gutiérrez-Diez, Miguel Ángel López-Marcos, Julia Martínez-Rodríguez, Jose Russo

**Affiliations:** 1 Department of Economic Theory and IMUVA, Faculty of Economics, Avda. Valle Esgueva 6, University of Valladolid, Valladolid, 47011 Spain; 2 Department of Applied Mathematics and IMUVA, Faculty of Science, University of Valladolid, Paseo de Belén 7, Valladolid, 47011 Spain; 3 Department of Applied Economics and IMUVA, Faculty of Economics, University of Valladolid, Avda. Valle Esgueva 6, Valladolid, 47011 Spain; 4 Director of the Breast Cancer Research Laboratory, Fox Chase Cancer Center, 333 Cottman Avenue, Philadelphia, 19111-2497 PA USA

**Keywords:** Optimal control problem, Time valuation factor, System of difference equations, Chronic myeloid leukemia, Hematopoiesis, Imatinib therapy

## Abstract

**Background:**

The mathematical design of optimal therapies to fight cancer is an important research field in today’s Biomathematics and Biomedicine given its relevance to formulate patient-specific treatments. Until now, however, cancer optimal therapies have considered that malignancy exclusively depends on the drug concentration and the number of cancer cells, ignoring that the faster the cancer grows the worse the cancer is, and that early drug doses are more prejudicial. Here, we analyze how optimal therapies are affected when the time evolution of treated cancer is envisaged as an additional element determining malignancy, analyzing in detail the implications for imatinib-treated Chronic Myeloid Leukemia.

**Methods:**

Taking as reference a mathematical model describing Chronic Myeloid Leukemia dynamics, we design an optimal therapy problem by modifying the usual malignancy objective function, unaware of any temporal dimension of cancer malignance. In particular, we introduce a time valuation factor capturing the increase of malignancy associated to the quick development of the disease and the persistent negative effects of initial drug doses. After assigning values to the parameters involved, we solve and simulate the model with and without the new time valuation factor, comparing the results for the drug doses and the evolution of the disease.

**Results:**

Our computational simulations unequivocally show that the consideration of a time valuation factor capturing the higher malignancy associated with early growth of cancer and drug administration allows more efficient therapies to be designed. More specifically, when this time valuation factor is incorporated into the objective function, the optimal drug doses are lower, and do not involve medically relevant increases in the number of cancer cells or in the disease duration.

**Conclusions:**

In the light of our simulations and as biomedical evidence strongly suggests, the existence of a time valuation factor affecting malignancy in treated cancer cannot be ignored when designing cancer optimal therapies. Indeed, the consideration of a time valuation factor modulating malignancy results in significant gains of efficiency in the optimal therapy with relevant implications from the biomedical perspective, specially when designing patient-specific treatments.

## Background

The design of optimal therapies to fight several illnesses, among them mainly cancer, is an important research in the field of Biomathematics. As a result, the textbooks and articles published during the last 15 years dealing with this subject are numerous (see for instance [1–3] or the textbooks [4–9]; for the specific case of cancer see [10–18] and the references provided by these authors). Basically, an optimal therapy problem is a control problem consisting of: first, a set of difference/differential equations describing the biological dynamics of the disease under the specified treatment; and second, an objective function measuring the malignancy of the treated cancer and that must be minimized. By solving this optimal control problem it is possible to find the optimal therapy, that is, the drug doses that minimize the malignancy of the treated disease.

The philosophy behind a cancer optimal therapy model can be easily explained and interpreted in biomedical terms. The key fact is the possibility of describing the dynamic behavior of the treated cancer through a system of difference/differential equations. More specifically, this system of equations describes the evolution of the number of tumor and normal cells in the course of a treatment with a drug. In this system, the number of cancer and normal cells are a consequence of the administered drug concentration, which is a completely controllable variable. In simple words, the administered drug doses constitute the input of the problem, the number of cancer and normal cells being the output. Then, since the behavior of normal and cancer cells is a function of the administered drug dose, it becomes feasible to govern the number of cancer and normal cells according to an objective by adequately manipulating the drug doses. This is precisely the idea underlying a cancer optimal therapy model: to identify the drug doses that minimize the damages to health caused by cancer and drug toxicity, damages quantified by an objective function measuring treated cancer malignancy.

Until now, the mathematical formulation adopted to measure the malignancy of a particular status of the disease, whatever the evolution/duration of the disease may be, exclusively considers as malignancy elements the tumor size, given by the number of cancer cells, and the administered drug concentration measured in a specific instant (see for instance the references here provided [10–18]). From this perspective, a given number of cancer cells and a given drug dosage would always entail the same malignancy, independently of the moment of time in which these magnitudes have been observed, i.e., independently of how the disease evolved, when the cancer reached the measured size, and when the drug dose was administered. However, from the biomedical perspective, it is a well-established fact that the faster the cancer grows the worse the cancer is, i.e., that a cancer that reaches a given size earlier is worse than a cancer reaching the same size later (see for instance [19–22]). To take into account this malignancy factor, ignored by the literature, would require higher malignancy to be assigned to cancer cells at the beginning of the cancer and lower malignancy to those appearing afterwards, that is, a time-valuation factor weighting cancer size. On the other hand, it is also widely accepted by biomedical researchers and practitioners that drugs are not totally eliminated by patients and remain in their bodies, and that, subsequently, a given drug dose entails more negative effects if it is administered at the beginning of treatment than at the end (see [23–26]). As happened with cancer size, to evaluate malignancy associated to drug doses, it would also be necessary to consider a time-valuation factor weighting them, and assigning higher malignancy to the initial drug doses.

The higher malignancy of early developed cancer can also be concluded from the heuristic perspective. On this point and as is obvious, one of the main questions to arise in our research is how to measure treated cancer malignancy and its evolution in time. To do this, we propose to measure the malignancy of treated cancer through the slope of the cancer survival curve in absolute value, since, according to survival theory, it represents the contribution of each instant in time to bring about death as a consequence of the considered cancer. The typical survival curves for cancer are markedly strictly convex, something implying decreasing slopes in absolute values and therefore decreasing malignancy in time. This happens for practically all treated cancers, among them colorectal ([27]), pancreatic ([28]), lung ([29]), hepatocellular ([30]), ovarian ([31]), breast ([32]), etc., and also for chronic myeloid leukemia (CML) ([33]).

In this respect, although this treated cancer malignancy/probability of dying needs not be decreasing in time for all treated cancers, this situation, implying non-convexity for the subsequent survival curve and non-decreasing malignancy, is the exception and not the rule. Indeed, to our knowledge, this happens only for some types of breast cancer and prostate cancer, the sole cancers presenting non-decreasing malignancy in time when diagnosed and treated (see [32], [34–36]).

In addition and as we will explain in detail in the following section, the design of realistic optimal therapy models requires the following aspects to be simultaneously formulated according to the empirical evidence: the mathematical behavior of the considered cancer; the particular biomedical effects of the administered drugs for this cancer; and the observed evolution in time of malignancy according to the cancer specific survival curve. All these interrelated aspects, whose output are the observed clinical data used in the calibration process, lead to a unique optimal therapy model, which is specific for the considered cancer.

In this respect, and as a representative case of the most generalized behavior of malignancy (convex survival curves and therefore malignancy decreasing in time), we focus on chronic myeloid leukemia and elaborate a specific model for imatinib-treated. We therefore consider the mathematical description of this disease arising from the observed clinical data, which imply specific interrelationships between the different types of cells, and specific drug effects compatible with convex survival curves (unequivocally observed for this type of cancer), and then a decreasing in time malignancy. From the heuristic perspective and besides the above mentioned clinical and medical data, this convex survival curve for imatinib-treated CML also suggests introducing a time valuation factor weighting cancer malignancy, and implying decreasing malignancy as cancer persists.

We study these questions for the standard formulation of optimal therapy models. More specifically, we formulate a modified discrete version of the ordinary differential equation model of Chronic Myeloid Leukemia dynamics proposed in [15], incorporating a daily schedule in the dynamics: since our purpose is to replicate the observed evolution of CML and to extract clinical conclusions, and given that in the empirical literature on CML and in clinical practice the parameters and the variables involved in the model are measured in per day values, we opt to consider a difference equation model in which time is discrete and represents a sequence of days. Then, we calibrate the model parameters to mirror the observed behavior of the disease and introduce a time valuation factor that allows the malignancy elements associated to time and commented on above to be considered. We analyze the consequences of the introduction of this time valuation factor decreasing over time with the purpose of: first, clarifying whether or not this time dependent malignancy factor modifies the optimal therapies and the population of normal and cancer cells; and, second, quantifying these modifications.

This paper is organized as follows. First, we consider a discrete CML mathematical model, introducing an optimal therapy problem and analyzing it from a numerical point of view. After assigning values to the model parameters through the calibration of the model, which is carried out for an average patient with CML at advanced phase, we present the simulation and numerical results for the calibrated model. Finally, we briefly comment on the main conclusions of the research.

## Methods

### A mathematical model of treated CML

Taking into account [17], in [15] a continuous time model was introduced to analyze the global dynamics of CML. Here, in order to gain consistency in the analysis, comparisons with real data and simulations, we propose a discrete time version of that model incorporating a daily schedule in the dynamics, since in the empirical literature on CML and in clinical practice the parameters and variables involved in the model are measured in per day values.

**Fig. 1:**
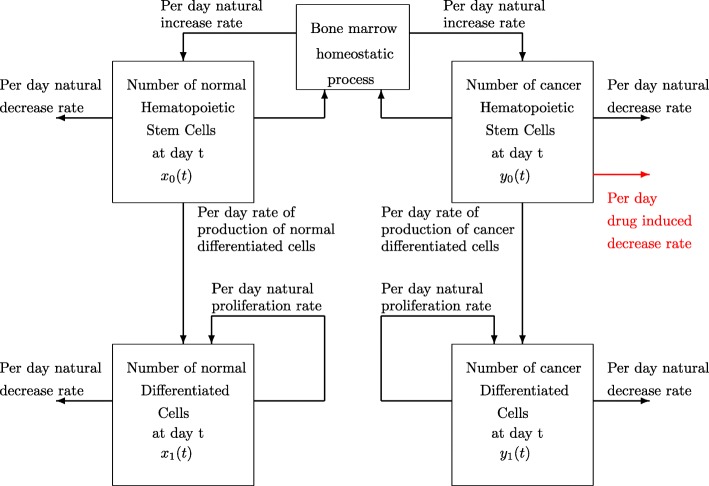
Day to day interactions among cancer and normal cells in CML

Our discrete time dynamic model of CML reproduces the biological interactions among the different types of normal and cancer cells observed in this disease, interactions represented in Fig. 1.

We start by considering two different populations: that of hematopoietic stem cells (HSC), and that of differentiated cells (DC). In addition, each of these populations is divided into normal cells and cancer cells. Then, denoting by *T* the treatment duration measured in days, at time instant *t*, *t*=0,1,…,*T*+1, it is possible to distinguish between four different populations: normal HSC, denoted by *x*_0_(*t*); cancer HSC, denoted by *y*_0_(*t*); normal DC, represented by *x*_1_(*t*); and cancer DC, denoted by *y*_1_(*t*).

The evolution over time of CML is described by a system of difference equations which incorporates the most relevant biomedical facts. First, the populations of all the considered types of cells naturally decrease at fairly constant rates. Upon this fact, let *d*_0_, *g*_0_, *d* and *g* be, respectively, the per day decrease rates of normal and cancer HSC, and normal and cancer DC. In addition, since DC are produced not only by proliferation of DC but also by HSC, it is necessary to distinguish between these two mechanisms of increase in the number of DC for both normal and cancer cells. In particular, let *d*_2_ and *g*_2_ be the per day rates at which normal and cancer DC proliferate and originate, respectively, normal and cancer DC; and let *r* and *q* denote the rates at which normal and cancer HSC produce normal and cancer DC, in this order. Finally, through the self-renewal process, normal and cancer HSC produce similar cells by division. Then, we let normal and cancer HSC divide at rates *n* and *m* per day, respectively. In this self-renewing activity, there underlies a homeostatic process that controls the proliferation of HSC. In this respect, the division of normal HSC, *x*_0_, is directed by homeostasis which is represented by a positive decreasing function *Φ*, depending on the total level (*x*_0_+*y*_0_), and given by

1 $$\begin{array}{@{}rcl@{}}  \Phi(x_{0}+y_{0})=1-\frac{x_{0}+y_{0}}{K}, \end{array} $$

where *K* represents the carrying capacity of bone marrow. In the same way, homeostasis for cancer cells, *y*_0_, is governed by a positive decreasing function *Ψ*, which depends on (*x*_0_+*αy*_0_), where *α*∈(0,1] measures the fall in the homeostatic efficiency due to the disease (see [10] and [16] for an analysis of this fall),

2 $$\begin{array}{@{}rcl@{}}  \Psi(x_{0}+\alpha y_{0})=1-\frac{x_{0}+\alpha y_{0}}{K}. \end{array} $$

This model incorporates the existence of nonlinear effects of imatinib treatment over a fixed period of time. Drug treatment is described by a positive time-dependent sequence *u*(*t*),*t*=0,1,…,*T*, which captures the drug dose, and given that there is a dosage limitation due to the drug’s toxicity, *u*(*t*) is supposed to be bounded in [0,*u*_*max*_], for *t*=0,1,…,*T*. The effects of imatinib treatment are introduced through nonlinear functions, which affect the lifetime of cancer cells, and imply their maximum effect only for an intolerable dosage. In [15], different scenarios were studied depending on the distinct effects of imatinib on the dynamics. That work concluded that the disease completely remits only when imatinib causes an additional mortality of cancer HSC. In addition, since imatinib is a targeted drug (see [37]), this is the case with highest plausibility from the biomedical point of view, so we limit our study to this situation. Drug effects are represented by the function *h*(*u*), which is a nonlinear increasing function satisfying *h*(0)=0 (this means that cancer HSC declines at baseline rate *g*_0_ without treatment). Clinical evidence also shows that patients rarely develop resistance to imatinib. Indeed, as the eight-year International Randomized Study of Interferon and STI571 (IRIS) trial concluded (see [38]), resistence to imatinib only appears for 6.75% of patients, and therefore we can assume that the function *h* only depends on the drug dose *u* and not on the elapsed treatment time. The behavior under treatment of CML is therefore described by the following system of difference equations:

3 $$\begin{array}{*{20}l} x_{0}(t+1)&=&x_{0}(t)+n\,\Phi \left(x_{0}(t)+y_{0}(t)\right) x_{0}(t)-d_{0}x_{0}(t), \end{array} $$

4 $$\begin{array}{*{20}l} x_{1}(t+1)&=&x_{1}(t)+{rx}_{0}(t)-{dx}_{1}(t)+d_{2}x_{1}(t), \end{array} $$

5 $$\begin{array}{*{20}l} y_{0}(t+1)&=&y_{0}(t)+ m\,\Psi \left(x_{0}(t)+\alpha y_{0}(t)\right) y_{0}(t)- g_{0}y_{0}(t)-h(u(t))y_{0}(t),\,\quad \end{array} $$

6 $$\begin{array}{*{20}l} y_{1}(t+1)&=&y_{1}(t)+{qy}_{0}(t)-{gy}_{1}(t)+g_{2}y_{1}(t), \end{array} $$

for *t*=0,1,…,*T*.

The system must be completed with the initial values for normal and cancer HSC and DC *x*_0_(0), *x*_1_(0), *y*_0_(0),*y*_1_(0).

### The optimal therapy problem and the time valuation factor

In this section we consider an optimal therapy problem where the objective is to find the therapy *u*^∗^(*t*), *t*=0,1,…,*T*, minimizing the malignancy of the disease under treatment. Then, the set of admissible controls is given by

$$\begin{array}{@{}rcl@{}} {\mathcal{U}}=\left\{\{u(t)\}_{t=0}^{T} \,\vert \, \, 0\le u(t) \le u_{max},\, t=0,1,\ldots, T \right\}. \end{array} $$

Concerning the objective function measuring malignancy which must be minimized, there is not in the literature any consideration of the higher malignancy associated with quick development of cancer and early drug administration. These functions measuring malignancy adopt different specifications (for instance, [18] considers quadratic terms representing the nonlinear costs of the treatment; [15] assumes quadratic terms to measure the nonlinear costs of both the treatment and the malignancy of the cancer HSC and DC levels; and [14] assumes linear costs for cancer cells and quadratic terms to measure treatment toxicity). However, and as commented on above, no valuation of time appears. Here we formulate the alternative objective function

7 $$\begin{array}{@{}rcl@{}}  N(u)=\sum_{t=0}^{T} \rho^{t}\left[u^{2}(t)+y_{0}^{2}(t)+y_{1}^{2}(t)\right]+\rho^{T+1}\left[y_{0}^{2}(T+1)+y_{1}^{2}(T+1)\right], \end{array} $$

where *ρ*∈(0,1] is a parameter measuring the increase of malignancy of early cancer development and drug administration. Case *ρ*=1 corresponds to the situation presented in the literature until now, which assumes that time does not affect either the malignity of cancer cells or the toxicity of the drug treatment. However, different values of *ρ*∈(0,1) highlight the important role of time. In this respect, and given that a higher malignancy is associated with both early cancer growth and early drug administration, we introduce such a time valuation malignancy factor, decreasing over time, that affects the objective function in the optimal therapy problem as a whole. This is consistent with the observed evidence for treated CML (see [39]), obviously the only existing situation of the disease for which data exist. Indeed, most empirical survival analyses show decreasing rates of mortality as treated cancer persists, i.e., higher mortality rates at the beginning of the disease than in subsequent dates. This decreasing rate of mortality over time would also suggest introducing a time valuation factor weighting cancer malignancy, and implying decreasing malignancy as cancer persists.

Summing up, we propose to solve and simulate the following optimal therapy problem:

$$\begin{array}{@{}rcl@{}}  \min_{\{u(t)\}_{t=0}^{T}\in\, {\mathcal{U}}} N(u) \end{array} $$

subject to the difference Eqs. (3)-(6) and

$$\begin{array}{@{}rcl@{}} &&x_{0}(t)\ge 0,\, x_{1}(t)\ge 0,\, y_{0}(t)\ge 0,\, y_{1}(t)\ge 0,\qquad t=0,1,\ldots,T+1,\\ && x_{0}(0),\, x_{1}(0),\, y_{0}(0),\, y_{1}(0), \qquad \text{initially given.} \end{array} $$

### Numerical solution and computational procedure

As usually happens with optimal control problems in Biomedicine, our optimal therapy problem does not have an algebraic solution. This compels us to numerically solve and simulate the model. Here, we consider an indirect method. The starting point is the derivation of the necessary conditions for the optimal control *u*^∗^(*t*), *t*=0,1,…,*T*. To this end, we use the discrete Maximum Principle by means of the Lagrangian function (see [40]), which involves: the system of difference equations for the state variables describing the dynamics of CML with the associated initial conditions; the system of difference equations for the Lagrange multipliers with the corresponding final conditions; and the nonlinear equation for the control variable.

We denote the Lagrange multipliers as *p*_1_(*t*),*p*_2_(*t*),*p*_3_(*t*) and *p*_4_(*t*), *t*=1,…,*T*+1, satisfying the following adjoint equations (where we substitute expressions (1) and (2) for functions *Φ* and *Ψ*, respectively),

8 $$\begin{array}{*{20}l} &&p_{1}(t)=p_{1}(t+1)\left[ 1-d_{0}+n\left(1-\frac{2x_{0}(t)+y_{0}(t)}{K}\right)\right]+ \end{array} $$

9 $$\begin{array}{*{20}l} &&\hspace{.5cm} p_{2}(t+1)r-p_{3}(t+1)\frac{m}{K} y_{0}(t),\\ &&p_{2}(t)=p_{2}(t+1)\left[1-(d-d_{2})\right], \end{array} $$

10 $$\begin{array}{*{20}l} &&p_{3}(t)=2\rho^{t}y_{0}(t)-p_{1}(t+1)\frac{n}{K}x_{0}(t)+ \end{array} $$

11 $$\begin{array}{*{20}l} &&\hspace{.5cm} p_{3}(t+1)\left[1-g_{0}-h(u(t))+m\left(1-\frac{x_{0}(t)+2\alpha y_{0}(t)}{K}\right)\right]+p_{4}(t+1)q,\\ &&p_{4}(t)=2\rho^{t}y_{1}(t)+p_{4}(t+1)\left[1-(g-g_{2})\right], \end{array} $$

for *t*=1,…,*T*, and the corresponding final conditions,

12 $$\begin{array}{*{20}l} p_{1}(T+1)&=&0, \end{array} $$

13 $$\begin{array}{*{20}l} p_{2}(T+1)&=&0, \end{array} $$

14 $$\begin{array}{*{20}l} p_{3}(T+1)&=&2\rho^{T+1}y_{0}(T+1), \end{array} $$

15 $$\begin{array}{*{20}l} p_{4}(T+1)&=&2\rho^{T+1}y_{1}(T+1). \end{array} $$

With respect to the stationary condition for the control, for *t*=0,1,…,*T*, we have that *u*^∗^(*t*) is the solution of

16 $$\begin{array}{@{}rcl@{}}  2\rho^{t}u(t)-p_{3}(t+1)h^{\prime}(u(t))y_{0}(t)=0,\quad \text{if}\quad u^{*}(t)\in(0,u_{max}), \end{array} $$

otherwise,

17 $$\begin{array}{*{20}l} u^{*}(t)=u_{max},\quad &\text{if}&\quad 2\rho^{t}u(t)-p_{3}(t+1)h^{\prime}(u(t))y_{0}(t)>0, \end{array} $$

18 $$\begin{array}{*{20}l} u^{*}(t)=0,\quad &\text{if}&\quad 2\rho^{t}u(t)-p_{3}(t+1)h^{\prime}(u(t))y_{0}(t)<0. \end{array} $$

In addition, the Eqs. (3)-(6) and the corresponding initial conditions for the state variables must be satisfied.

For the numerical solution of this problem, we employ a forward-backward sweep method (see [41]) by writing an algorithm in Matlab^®^ with the procedure specified in the Appendix.

We implement this numerical method for a clinical case. To this end, it is previously necessary to calibrate the parameters, i.e., to assign a value to the parameters in the system of difference equations providing the solution. This calibration will be carried out in the following section on the basis of the available recent biomedical data, whenever possible, and of the dynamic properties of the model.

### Assigning values to the model parameters: the calibration of the model

Rather than assigning values to the parameters on the basis of statistical estimation procedures (see for instance [42] or [43]), we will adopt the calibration approach (see for instance [15] and [18]) using when possible real data obtained from biomedical evidence. This procedure has an immediate advantage: In principle and with the only restriction of data availability, the calibration of the model could be done for each particular patient, a very interesting question from the clinical perspective since it opens up the possibility of designing personalized therapies.

Here we carry out an update and extension of the calibration process followed in [15]. These authors assign values for the bone marrow capacity and for the division, decline and production rates of normal cells on the basis of the biomedical data provided by Michor et al. [17], the remaining parameter values being fixed according to mathematical criteria. Here we consider more recent biomedical empirical evidence on a greater set of parameters, not only those in [15], but also for the division and mortality rates of cancer HSC and cancer DC, maximum recommended daily dose of imatinib, effectiveness of imatinib, and days to complete hematological response. As reference, we consider an average patient with CML at advanced phase, who is totally recovered after treatment with imatinib. According to [39], this happens in 38% of the cases.

As commented on before and for the sake of reproducing realistic behaviors of the treated disease, the values of the parameters in the model are calibrated on the basis of the more recent and reliable available biomedical data (see [33, 44–47]). Regarding division and mortality rates for normal HSC, we take the estimates provided by [45], which fix a per day division rate $n=\frac {1}{280}\approx 0.0036$ and a mortality rate $d_{0}=\frac {1}{2000}=0.0005$. In that work we also find the steady number of normal HSC $\overline {x}_{0}=11000$, from which we can compute the estimate of the per-day carrying capacity of bone marrow *K* consistent with the model: since the dynamics of normal HSC is ruled by (3), at the steady state for a healthy patient

$$\begin{array}{@{}rcl@{}} 0=\frac{1}{280}\left (1-\frac{11000}{K}\right)-\frac{1}{2000}, \end{array} $$

and then we take *K*=12791.

The rate of generation of normal DC from normal HSC is taken as *r*=10^11.5^≈3.1623e+11 (see [46]).

Concerning the value of the mortality rate of DC, it was calculated on the basis of the lifetimes of their different types and respective percentages. As collected by [47], these lifetimes and percentages are roughly the following: Neutrophils (62%), 6 hours; Eosinophils (2.3*%*), 8 days; Basophils (0.4*%*), 2 hours; Lymphocytes (30%), 1.5 days; and Monocytes (5.3*%*), 4 hours. These data imply an average lifetime of 0.8 days, and therefore a per day mortality rate of $d=\frac {1}{0.8}=1.25$. Regarding the mortality rates of cancer HSC and DC and according to the recent research by [44] showing loss of apoptosis for cancer cells in CML, we fix *g*_0_=0.0003<*d*_0_ and *g*=1.1<*d*. For the parameters *α*, *q*, *g*_2_,*d*_2_ and *g*, there are no empirical observations. However, in accordance with the results in [15], given that the stability and dynamic properties of the model do not depend on these parameters, we can fix arbitrary values for them. In particular, we assign the values *α*=0.1, *q*=10^11.5^=*r* and *d*_2_=0.25. Work in [44] also finds that the division rates for cancer HSC and DC in CML are higher than for normal HSC and DC; but there are no empirical observations concerning this rate. In this respect, having accepted that *m*>*n* and *g*_2_>*d*_2_ and given that the asymptotic behavior of the solution of the uncontrolled problem does not depend on the value of *m* and *g*_2_ (as in the continuous model [15]), we fix a value for *m* slightly greater than *n*. In particular, we assign the values *m*=0.0037>*n* and *g*_2_=0.5>*d*_2_. In any case, as we shall see, the calibration of the function *h*(*u*) is conditioned by the assumed set of values: the adoption of other values would simply imply a re-calibration of *h*(*u*). Table 1 collects all the previously calibrated values for the parameters.

**Table 1:** Calibrated values for the parameters in the model

Parameter	Description	Value	Units
*n*	Normal HSC division rate	0.00357	/day
*m*	Cancer HSC division rate	0.0037	/day
*d*_0_	Normal HSC mortality rate	0.005	/day
*g*_0_	Cancer HSC mortality rate	0.0003	/day
*r*	Normal DC production rate	10^11.5^	/day
*q*	Cancer DC production rate	10^11.5^	/day
*d*	Normal DC mortality rate	1.25	/day
*d*_2_	Normal DC proliferation rate	0.25	/day
*g*	Cancer DC mortality rate	1.1	/day
*g*_2_	Cancer DC proliferation rate	0.5	/day
*K*	Carrying Capacity of Bone Marrow	12791	HSC/day

With respect to the drug treatment, the maximum recommended daily dose *u*_*max*_ is 800 mg/day (see [39] and [48]). Calibration of the function *h*(*u*), representing the drug effects, is as follows. First, concerning its qualitative properties, we impose that: *h*(0)=0;*h*(*u*) must be non linear; and verifying $\frac {dh(u)}{du}>0$ and $\frac {d^{2}h(u)}{du^{2}}<0$. In biomedical terms, we are assuming well established facts (see [17]): that if no drug is administered there are no drug effects; that imatinib effects are non linear; that higher doses imply higher effectiveness; and that these gains of effectiveness are decreasing. Several mathematical expressions capturing these generic features are possible (see for instance [15] or [3]). In this respect, and given that there exists empirical evidence suggesting a logarithmic relationship between biological responses and their causing factors (see [49–51] and the references provided by these works), we assume a logarithmic expression for *h*(*u*):

19 $$\begin{array}{@{}rcl@{}}  h(u)=\overline{h} \ln\left(1+u\right). \end{array} $$

In this case, the nonnegative root of (16) satisfies

20 $$\begin{array}{@{}rcl@{}}  u(t)=\frac{1}{2}\left(-1 +\sqrt{1+{\frac{2\,\overline{h\,}p_{3}(t+1)\, y_{0}(t)}{\rho^{t}}}}\right). \end{array} $$

Finally, in order to fit the value of $\overline {h}$ in (19), we impose the fulfillment of the following biomedical facts, collected in [39]:

- A fixed imatinib dose of 600 mg/day gets a complete hematological response, i.e., the total disappearance of cancer HSC and DC, and the recovery of the number of normal HSC and DC to their steady healthy values in about 36 months.
- The gain of effectiveness after increasing the drug doses from 400 mg/day to 600 mg/day is about 1.17.

Note that function *h* in (19) satisfies $\frac {h(u=600)}{h(u=400)}=1.07$, a reasonable approximation to the empirical evidence. On the other hand, we have simulated the model with the constant dose *u*(*t*)=600, *t*=0,1,2,…, and starting with the initial data:

$$\begin{array}{@{}rcl@{}} x_{0}(0)=10^{4},\qquad x_{1}(0)=10^{11},\qquad y_{0}(0)=10^{3},\qquad y_{1}(0)=10^{10}, \end{array} $$

representative of CML at advanced phase. We have observed that $\overline {h}=0.00455$ provides a close approximation to a complete hematological response after 36 months. It is worth noting again that, since we close the calibration process with the calibration of *h*(*u*), different values for the previously calibrated parameters would lead to a new expression for *h*(*u*) consistent with all the observed biomedical data (see [17, 39]). From this perspective, our calibration of $\overline {h}$, based on simulations of the model, lies on the same principles as those in [52].

Finally, concerning *ρ*, the parameter measuring the decrease in malignancy of the treated disease as time passes, we calibrate a value *ρ*<1 on the basis of the survival analyses for CML carried out by [33]. More specifically, we analyze this parameter assuming that it measures the decrease in the slope of the survival curve, i.e., the decrease in the death density. As this slope represents the contribution of each lapse of time to the probability of dying (see [10]), decreases in this contribution with time can be interpreted as decreases in the malignancy of the disease. In geometrical terms, the assumed decrease in malignancy must cause a decrease in the absolute value of the slope of the survival curve, that is, it must imply a convex survival curve. Empirically, this convexity is a universal feature of survival curves in cancer, something that supports our approach. This convexity has also been found in CML. Indeed, survival curves for treated CML in [33] (which we denote by *S*(*t*)) are markedly convex. In that paper, the authors carry out an exhaustive survival analysis of patients with CML under different treatments, concluding that the slope changes from significant negative initial values to a final value of zero. Since according to our argument

$$\begin{array}{@{}rcl@{}} \frac{S^{\prime}(t_{i})}{S^{\prime}(t_{f})}= \rho^{t_{i}-t_{f}}, \end{array} $$

this fact justifies any value for *ρ* lower than 1.

## Results and numerical simulations

### Uncontrolled/untreated dynamics

Once the parameters have been calibrated, the next step is to study the dynamics of the model. First of all, in order to test its biological feasibility, we consider the uncontrolled (or untreated) situation, that is, we analyze the time evolution of cells described by (3)-(6) when *u*(*t*)=0, *t*=0,1,… (remember that *h*(0)=0).

**Fig. 2:**
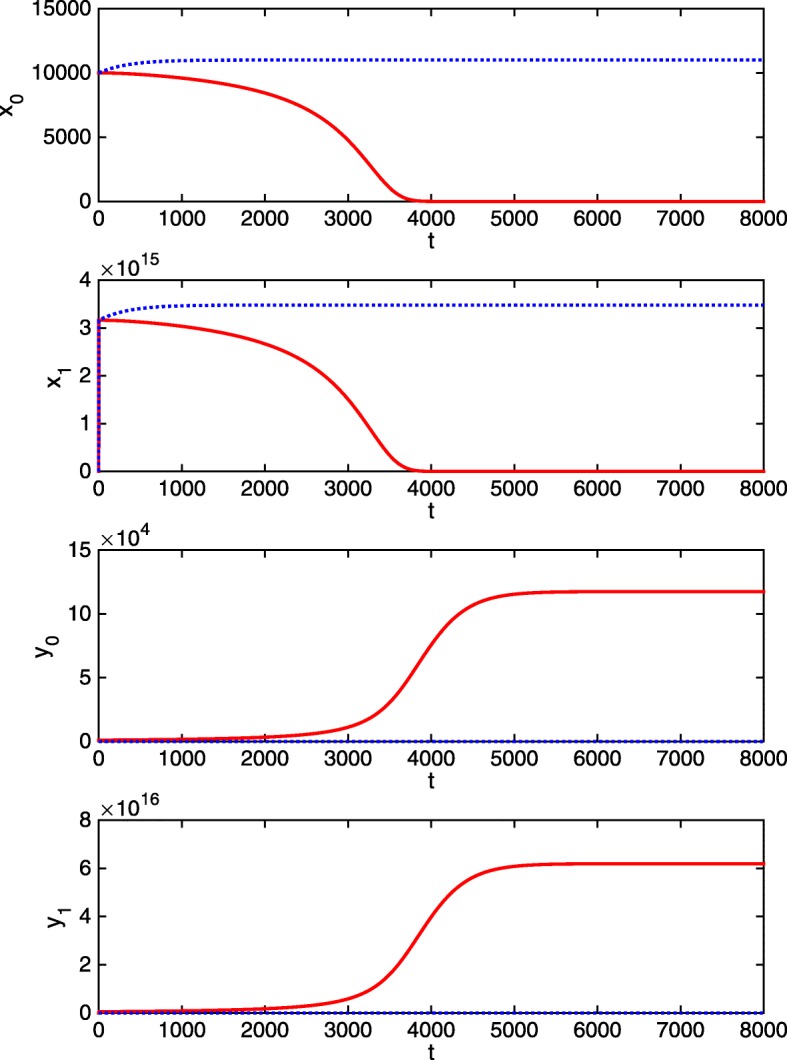
Uncontrolled dynamics. Time evolution in days (x-axis) of cells. Safe case: dashed line. Blast cases: solid line

As a first experiment we consider the evolution of normal HSC and normal DC when the disease is not present, i.e., when initially *y*_0_(0)=0 and *y*_1_(0)=0. Figure 2 shows the evolution of the different cells, with dashed line, when the initial levels of normal cells are fixed as *x*_0_(0)=10^4^, *x*_1_(0)=10^11^.

Note that the solution is attracted towards the safe steady state of the system (as in the continuous model in [15]):

$$\begin{array}{@{}rcl@{}} \overline{x}_{0}=K\left(1-\frac{d_{0}}{n}\right)=11000,\; \overline{x}_{1}=\frac{r}{d-d_{2}}\overline{x}_{0}\approx 3.48\text{e}+15,\; \overline{y}_{0}=0,\; \overline{y}_{1}=0. \end{array} $$

Since according to the medical literature (see [39]) the non-existence of cancer HSC and DC, and the recovery of the number of normal HSC and DC in the blood are the crucial values to ascertain the remission of the disease –complete hematological response–, this safe steady state is the reference to determine the overcoming of CML.

Now, assume that there exist cancer cells. Figure 2 also shows this solution, with solid line, obtained with the previous initial levels of normal cells, and when the initial values of cancer cells are *y*_0_(0)=10^3^ and *y*_1_(0)=10^10^.

In accordance with biomedical evidence, the solution is attracted towards the blast steady state, which corresponds to the total prevalence of cancer cells and the complete disappearance of normal cells (again, as in the continuous model in [15]):

$$\begin{array}{@{}rcl@{}} \overline{x}_{0}=0,\; \overline{x}_{1}=0,\; \overline{y}_{0}=\frac{K}{\alpha}\left(1-\frac{g_{0}}{m}\right)\approx 117539,\; \overline{y}_{1}=\frac{q}{g-g_{2}}\overline{y}_{0}\approx 6.19\hbox {e}+16. \end{array} $$

As is logical, under the absence of treatment, this is the steady state reached when *y*_0_(0)>0.

### Controlled/treated dynamics

#### Non truncated treatment

**Fig. 3:**
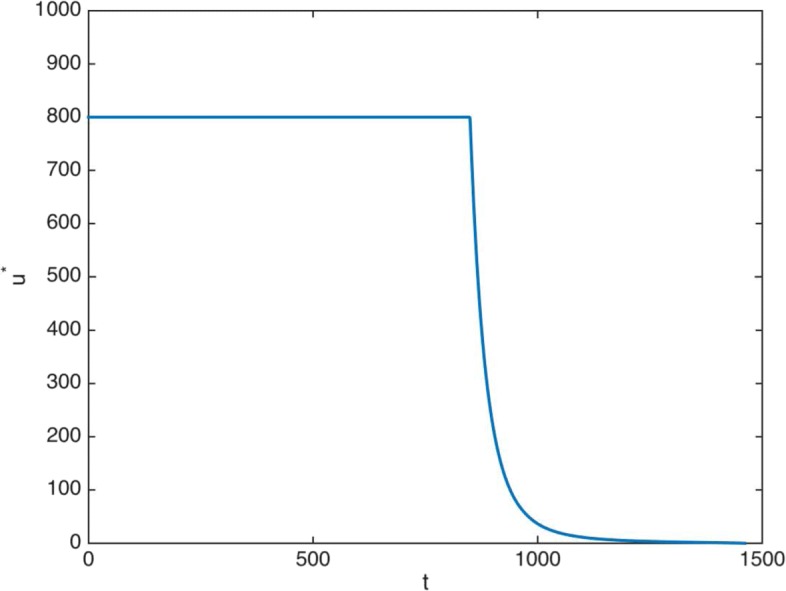
Non truncated treatment. Optimal therapy without time dependence in the objective function (*ρ*=1). Time in days (x-axis)

The above considered initial values *y*_0_(0)=10^3^ and *y*_1_(0)=10^10^, representative of CML at advanced phase, constitute just the initial situation that we analyze under drug treatment. Firstly, we simulate the case *ρ*=1: the objective function (7) does not depend on time. We assume that the treatment has a duration of 4 years, according to [26], a usual observed length for imatinib treatments in CML at advanced phase. Now, we implement the forward-backward sweep method described in the Appendix. As initial guess for the control we chose *u*(*t*)=*u*_*max*_, *t*=0,1,…*T*. Figure 3 presents the solution of the optimal control problem that appears. We observe an initial time period (850 days) of maximum daily dose, followed by a fast decay.

**Fig. 4:**
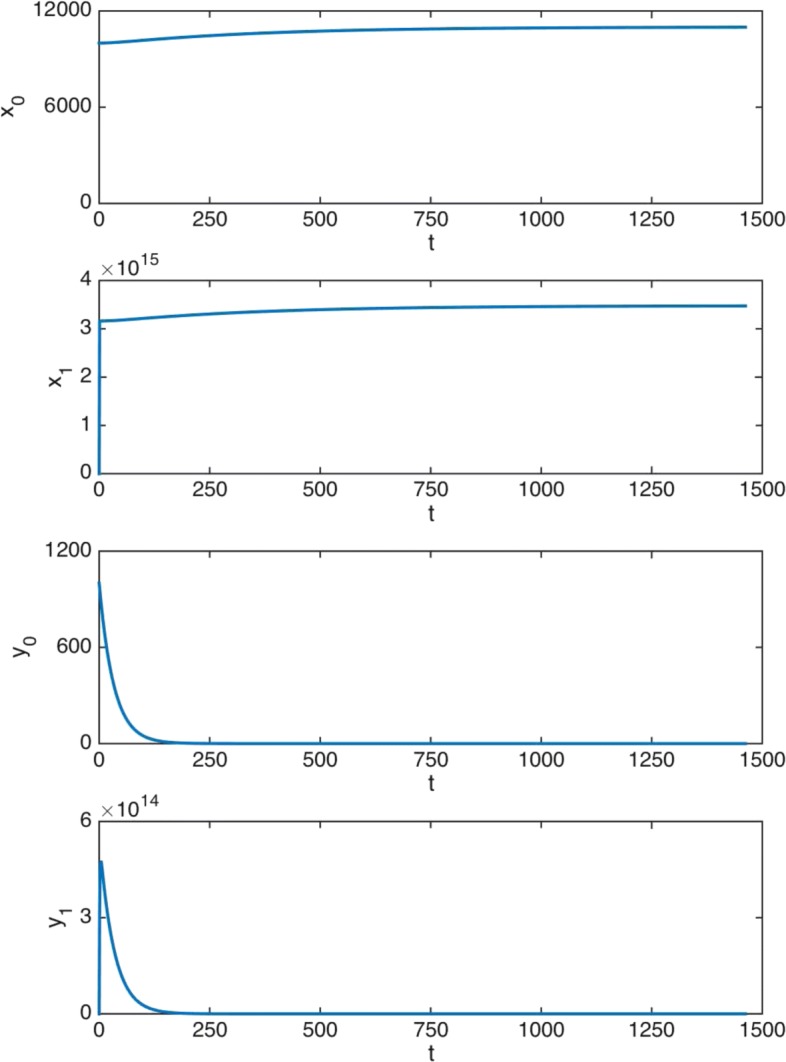
Non truncated treatment. Time evolution in days (x-axis) of cells in the optimal therapy without time valuation in the objective function (*ρ*=1)

The cell dynamics of the optimal solution is presented in Fig. 4: the disease remits, since the cancer HSC and cancer DC continuously decrease and are almost eliminated by drug optimal dosages reaching undetectable values.

We now consider a time valuation factor less than 1. A value of *ρ*<1 reflects the malignity of cancer cells and the toxicity of the drug treatment in the model. However, for the numerical computation of the optimal control solution, some values of *ρ* are not valid. Note that, when *ρ*<1, *ρ*^*t*^ decays to zero as *t* increases, and the smaller the factor, the faster the decay. This implies that if *ρ* is sufficiently small, the division in (20) cannot be implemented in the computer due to arithmetic underflow. In this sense, the smallest value that can be considered is *ρ*=0.65. For the feasible values of *ρ*, a qualitatively similar control solution to the case *ρ*=1 can be observed: an initial time period of maximum daily dose followed by a fast decay.

**Fig. 5:**
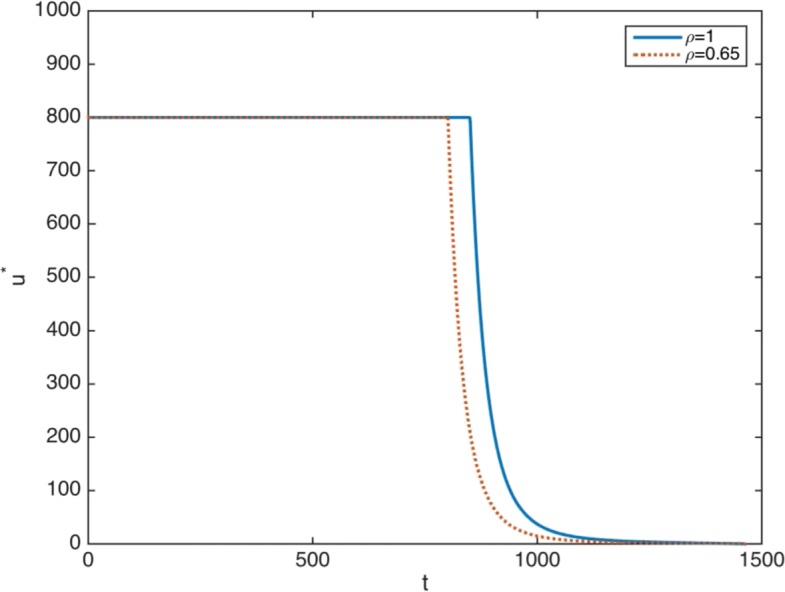
Non truncated treatment. Optimal therapy. Solid line: without time valuation (*ρ*=1). Dashed line: with time valuation (*ρ*=0.65). Time in days (x-axis)

Table 2 provides some representative data of the treatment for different values of *ρ*. First, the duration of the maximum dose, represented by *T*_*max*_. Second, the accumulative total dose, represented by *U*_*Tot*_. For these two variables we observe quantitative differences: both values decrease as *ρ* decreases. Consequently, we consider the limit value *ρ*=0.65 particularly worth studying, since this is the most extreme case with the most pronounced results. In Fig. 5, the computed optimal control with time valuation (dashed line) and without time valuation (solid line) are plotted.

**Table 2:** Representative parameters of the control solution depending on the discount factor *ρ*

*ρ*	1	0.9	0.8	0.7	0.65
*T*_*max*_ (days)	850	829	816	806	801
*U*_*Tot*_ (mg)	715372	696192	685644	677046	673026

*T*_*max*_: duration of the maximum dose. *U*_*Tot*_: accumulative total dose

It is important to note that the introduction of a time valuation factor implies a noticeable decrease in the optimal drug doses. As Fig. 5 shows, the main features of this descent when *ρ*=0.65 are the following: first, the duration of the maximum dose of 800 mg/day is 801 days, that is, the maximum amount of imatinib per day is retired 49 days before; second, when the optimal dose is lower than 800 mg/day, it is always significantly lower than the optimal dose when *ρ*=1; and third, the accumulated decrease in the administered drug is high. The treatment consists of 715372 mg when *ρ*=1, while it is of 673026 mg when *ρ*=0.65. This is a total saving of 42346 mg along the whole period of treatment: 6% of the total dose.

It is worth providing some intuitive ideas on the underlying biomedical mechanisms causing such drug dose descents, since they constitute one of the main findings of the research. As commented on in the previous sections, *ρ*<1 means that, given fixed numbers of cancer cells and drug dose, malignancy is higher if these numbers of cancer cells and the administered drug dose occur at the beginning of the treated disease. Consequently, other things being equal, malignancy decreases as time passes, and our optimal therapy problem takes account of this fact by assigning lower malignancy to given numbers of cancer cells if they happen later. The logical outcome is the administration of optimal lower drug doses as time passes in comparison with the optimal therapy when *ρ*=1. This is clear when we reach day 801: since both therapies (when *ρ*=1 and there is no time valuation, and when *ρ*=0.65 and there is time valuation) have previously administered the maximum doses every day, the drug dose and the number of cancer cells at day 802 will be the same for both alternatives; however, optimal therapy when *ρ*=0.65 interprets that this number implies lower malignancy and requires a lower drug dose than that administered by the optimal therapy when *ρ*=1, which has not assigned any decrease in malignancy. This mechanism continues from day 802 onwards, and the result is the identified decreases in the imatinib doses. The key point is now the associated relative increases in the numbers of cancer cells for the optimal therapy when *ρ*=0.65. Indeed, since the drug doses are lower, the numbers of cancer cells when *ρ*=0.65 must be higher than when *ρ*=1. Therefore, if these increases in the number of cancer cells compensate the decrease in the assigned malignancy, the optimal therapy could imply the need for higher imatinib doses for *ρ*=0.65. In this respect, we obtain another crucial result showing that this does not happen.

**Fig. 6:**
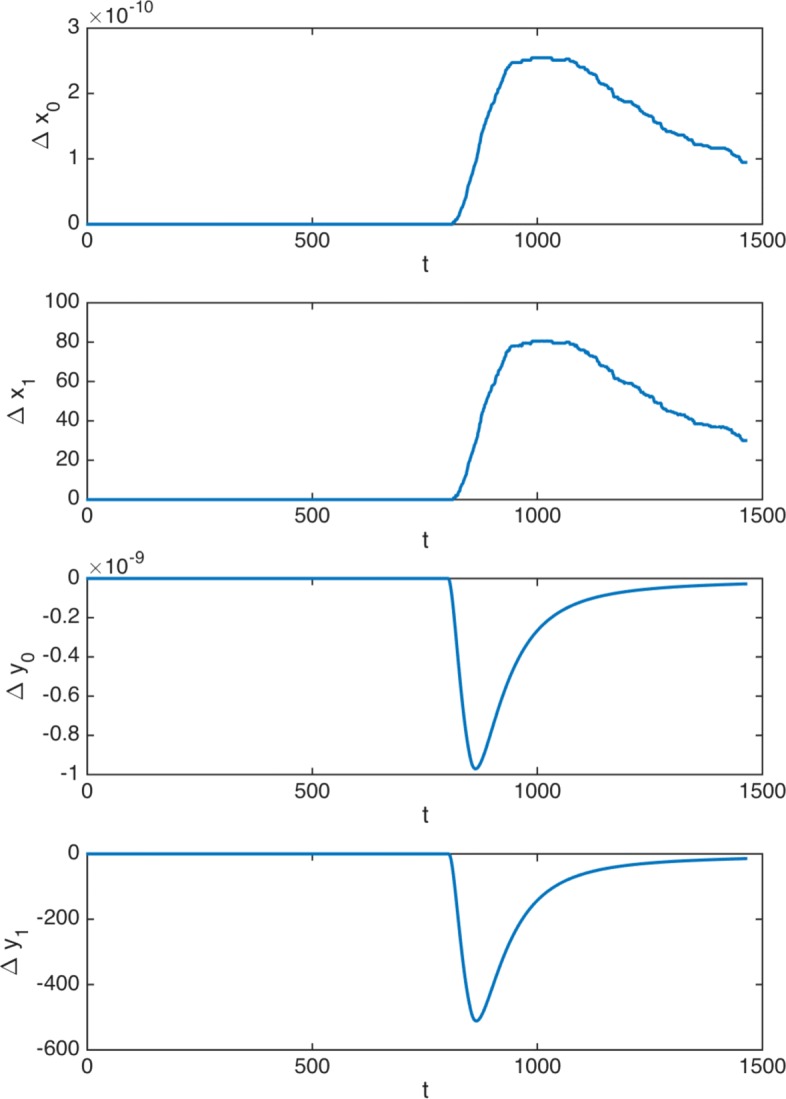
Non truncated treatment. Differences between the number of cells obtained with *ρ*=1 and *ρ*=0.65. Time in days (x-axis)

Indeed, with respect to the number of cells at each moment, we observe a very similar behavior to that depicted in Fig. 4 for *ρ*=1. Figure 6 shows the differences between cells obtained with *ρ*=1 and *ρ*=0.65 (denoted by *Δx*_0_(*t*),*Δx*_1_(*t*),*Δy*_0_(*t*) and *Δy*_1_(*t*),*t*=0,1,…*T*+1).

Note that the levels of cells at the end of the treatment are practically indistinguishable. The introduction of a time malignancy factor *ρ*=0.65 implies a negligible increase in the number of cancer HSC, always lower than 2.77e−11, and lower than 9.71e−10 at the end of the treatment. Concerning cancer DC, the results are similar. In particular, the increase in the number of cancer DC when *ρ*=0.65 is always lower than 511.49, and lower that 14.60 at the end of the treatment. Since the number of normal DC is about 3.48e+15, this increase is less than a million-millionth percentage of the number of normal cancer cells, clinically undetectable and irrelevant (see [53]).

#### Truncated treatment

It is of interest to note that, in both cases and after an initial time period of maximum daily dose, a fast decay appears. Then, from the clinical point of view, it can be assumed that there exists an effective duration of treatment depending on a medical minimal dose. After this period, the daily dose is negligible and then it can be assumed that no more drug is given. Here, for the CML treatment, we consider that the minimal dose of imatinib is 8 mg, that is, 1% of the maximum daily dose. This truncated treatment is worth studying due to its clinical applicability. In this case we observe that the effective length of the therapy is 1138 days when *ρ*=1 and 1053 days when *ρ*=0.65. This represents an important decrease in the duration of the treatment, more specifically 85 days, almost 3 months less. In addition, the resulting treatment obtained with *ρ*=0.65 provides a plausible duration period of about 35 weeks for a successful therapy.

**Fig. 7:**
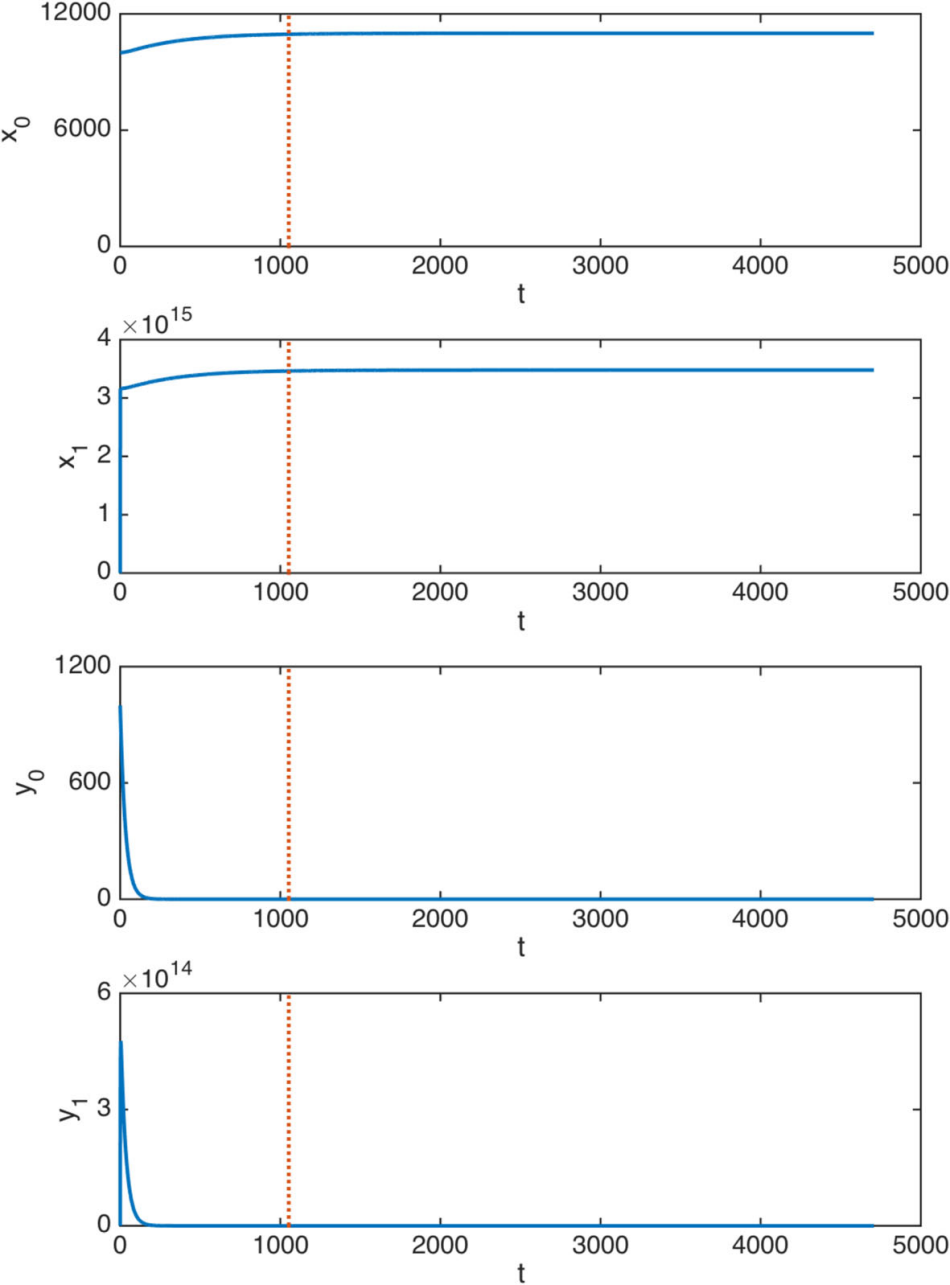
Truncated treatment. Time evolution in days (x-axis) of cells: effective 1053 days therapy (*ρ*=0.65), 10 years tracing

In the following experiment we control and keep track of the progress of the illness under this truncated treatment. Figure 7 shows the effectiveness of the optimal therapy obtained for *ρ*=0.65 over a period of 1053 days (effective duration of the treatment), which is illustrated here by a dashed vertical line. Note that, in this truncated version, the treatment consists of 672021 mg: we reduce the total dose by 1005 mg. If we now observe the evolution of cells over 10 years, the cell levels are 11000 normal HSC, 5.45e-10 cancer HSC, 3.48e+15 normal DC, and 288 cancer DC. Therefore, the number of normal cells corresponds to the safe equilibrium state and the number of cancer cells is negligible from the biomedical perspective.

Summing up, it can be concluded that, when *ρ*=0.65, the drug optimal doses are considerably lower and do not entail significant increases in the number of cancer HSC and cancer DC or in the disease duration. Therefore, the quantitative consequences of the consideration of a malignancy factor capturing the higher malignancy associated to early growth of cancer and drug administration are unequivocal: There is a gain of efficiency after the consideration of the time valuation factor. Indeed, since it allows more efficient therapies to be designed, the consideration of the existence of a time-valuation factor becomes very relevant to the design process of optimal therapies and cannot be ignored by practitioners and biomathematicians.

## Discussion and conclusions

The design of optimal therapies in cancer has been the subject of increasing research over recent years. Until now, the objective function has considered that malignancy exclusively depends on the drug concentration and the number of cancer cells. However, from the biomedical perspective, it is universally accepted that the faster the cancer grows the worse the cancer is, and that early doses are more prejudicial. To take into account these malignancy dimensions associated to the course of time, ignored by the literature but empirically observed in survival analyses, it is necessary to incorporate time-valuation factors into the objective function, assigning a higher malignancy to the initial periods. In this paper we study these questions for the standard formulation of optimal therapy models.

In particular, considering a discrete model of Chronic Myeloid Leukemia, we introduce a time valuation factor that allows the malignancy elements associated to time and commented on above, to be considered. Since clinical and biomedical data on CML are chosen in per day values, and the treatment and follow-up of the disease are carried out according to a daily schedule, this discrete time approach allows a better replication of the CML evolution to be obtained, easing the interpretation of the model and the extraction of clinical conclusions and recommendations. In addition, from the purely mathematical perspective, the use of difference equations instead of ordinary differential equations does not entail any loss of analysis capability (see [10], chapter 7, and the references therein). Moreover, we have verified that the dynamic behavior of the uncontrolled problem is just the same in both situations. In fact, the modeling of treated CML dynamics through a system of ordinary differential equations in continuous time needs of a discretization of time to be numerically solved, and this leads to a discrete model equivalent to the difference equations model we have considered.

### Conclusions

Our conclusions are unequivocal: the consideration of a time-valuation factor capturing the higher malignancy associated to early growth of cancer and drug administration allows more efficient therapies to be designed, and is then a very important element when designing cancer optimal therapies. More specifically, when this time valuation factor is incorporated into the objective function, not only the optimal drug doses are lower (a change in *ρ* from 1 to 0.65 involves 6% saving of the total dose), but also do not involve significant increases in the number of cancer cells or in the disease duration. The time valuation factor therefore cannot be ignored when designing cancer optimal therapies, since it results in significant modifications in the optimal therapy with relevant implications from the biomedical perspective, specially when designing patient-specific therapies. In this respect, given that the proposed optimal therapy model can be solved and simulated for any set of values for the parameters in Table 1, once we count on these values for a specific patient, it is straightforward to compute her/his personalized therapy as well as to calculate the subsequent reductions in the drug doses.

## Appendix

For the numerical experiments we employ the following forward-backward sweep method:

1. We start with an initial guess for *u*(*t*) at *t*=0,…,*T*.
2. With this initial iterant and making use of the initial conditions and equations (3)-(6), we forward compute the corresponding values of the state variables.
3. From the resulting values of the state variables and making use of equations (8)-(11) and the final conditions (12)-(15), we backward calculate the Lagrange multiplier variables.
4. By means of these approximations, we compute a new control variable following the procedure (16)-(18).
5. Finally, we iterate the procedure until convergence to the optimal control *u*^∗^(*t*), *t*=0,…,*T* (in practice, we stop when two consecutive iterations are close enough). Then, we generate the associated optimal values $x_{0}^{*}(t)$, $x_{1}^{*}(t)$, $y_{0}^{*}(t)$, and $y_{1}^{*}(t)$, *t*=0,…,*T*+1.

## Abbreviations

CML: Chronic myeloid leukemia
DC: Differentiated cells
HSC: Hematopoietic stem cells
